# Clinically relevant autistic traits predict greater reliance on detail for image recognition

**DOI:** 10.1038/s41598-020-70953-8

**Published:** 2020-08-28

**Authors:** Arjen Alink, Ian Charest

**Affiliations:** 1 University Medical Centre Hamburg-Eppendorf, Hamburg, Germany; 2 School of Psychology, Centre for Human Brain Health, University of Birmingham, Birmingham, UK

**Keywords:** Cognitive neuroscience, Perception, Object vision, Autism spectrum disorders

## Abstract

Individuals with an autism spectrum disorder (ASD) diagnosis are often described as having an eye for detail. But it remains to be shown that a detail-focused processing bias is a ubiquitous property of vision in individuals with ASD. To address this question, we investigated whether a greater number of autistic traits in neurotypical subjects is associated with an increased reliance on image details during a natural image recognition task. To this end, we use a novel reverse correlation-based method (feature diagnosticity mapping) for measuring the relative importance of low-level image features for object recognition. The main finding of this study is that image recognition in participants with an above-median number of autistic traits benefited more from the presence of high-spatial frequency image features. Furthermore, we found that this reliance-on-detail effect was best predicted by the presence of the most clinically relevant autistic traits. Therefore, our findings suggest that a greater number of autistic traits in neurotypical individuals is associated with a more detail-oriented visual information processing strategy and that this effect might generalize to a clinical ASD population.

## Introduction

Autism is a developmental disorder, now referred to as autism spectrum disorder (ASD), that manifests itself in a variety of forms. Diagnostic criteria for ASD include persistent deficits in social interactions and communication and repetitive patterns of behavior^1^. An intriguing aspect of ASD is that it is also associated with superior performance for tasks that involve the processing of visual detail. For example, ASD has been associated with superior performance for the embedded figures test^2–4^, which involves searching for a simple shape contained by a complex figure, and faster identification of the odd-man-out in cluttered displays^5–7^. A well-known, albeit unrepresentative, case of ASD-related enhanced processing of visual detail is the savant ability of Stephen Wiltshire, who is able to draw highly detailed urban landscapes after having seen his subject only briefly (https://www.stephenwiltshire.com).

In short, ASD has been associated with having an eye for detail. This, however, has been argued to come at the cost of a reduced ability to ‘see the big picture’ according to the influential weak central coherence (WCC) theory^8,9^. The original WCC formulation^10^ proposed that a bias towards processing details might underlie deficits in social functioning central to ASD^11^: a focus on details could cause individuals with ASD to miss socially meaningful cues that are global in nature, like facial expressions^12^. However, a string of studies finding no evidence for a relationship between perceptual measures of weak central coherence and measures of theory of mind and social skills^13–17^ led to a revised version of WCC^9^ suggesting that a bias towards processing details and social deficits might be two distinct aspects of ASD. Consequently, Happé and Frith^9^ pointed out that the future veracity of WCC critically depends on establishing a relationship between a detail-focused processing bias and real-life abilities and difficulties in ASD individuals. One way of supporting the feasibility of such a relationship would be to demonstrate that this bias is a ubiquitous property of vision in individuals with ASD.

ASD-related detail-focused processing has been initially supported by superior detail-focused abstract visual tasks, including the embedded figure task^3^ and the Navon task^17^. These early findings, however, are highly controversial given the fact that more recent studies have frequently been unable to replicate them^18^. An important endeavour of ASD research has been to determine if this detail-focused perceptual style is grounded in deviant low-level sensory processing in early sensory brain areas^19^. Despite the fact that several basic measures of visual sensitivity appear to be unaffected by ASD^16,20–23^, detail-focused processing has been associated with ASD in the context of face and object perception^20,22,24^. In addition, mixed evidence has been provided for ASD being related to enhanced sensitivity to high vs. low spatial frequency grating stimuli, as earlier studies found no such relationship^25,26^ while a more recent study by Kéïta et al.^20^ did find a relationship between ASD and enhanced sensitivity for high-spatial frequency grating stimuli.

Given the restricted and mixed nature of the current literature on ASD-related detail-focused processing, further research is needed to resolve whether such a bias represents a ubiquitous property of natural day-to-day image perception in individuals with ASD. Therefore, we here use a new reverse-correlation based ‘feature diagnosticity mapping’ paradigm to determine how the presence of autistic traits affects the relative contribution of low-level visual features to natural image recognition. Specifically, we used the 50-item Autism Spectrum Quotient (AQ) questionnaire for adults^24^ to split our neurotypical participants into a high- and low AQ group and assessed if image recognition depended more on high-spatial frequency stimulus features in the high AQ group. This approach relies on the notion that the comparison of individuals with many vs. few autistic traits can effectively model differences between individuals with and without a clinical ASD diagnosis^4,25–27^. In addition to testing for a relationship between ASD and detail-based image recognition, this approach is also used to test if previous reports of reduced gaze duration towards eyes^28–30^ and increased gaze durations for the central area of images^31^ can be related to image recognition, with individuals with ASD relying more on these aspects of images.

## Results

**Figure 1:**
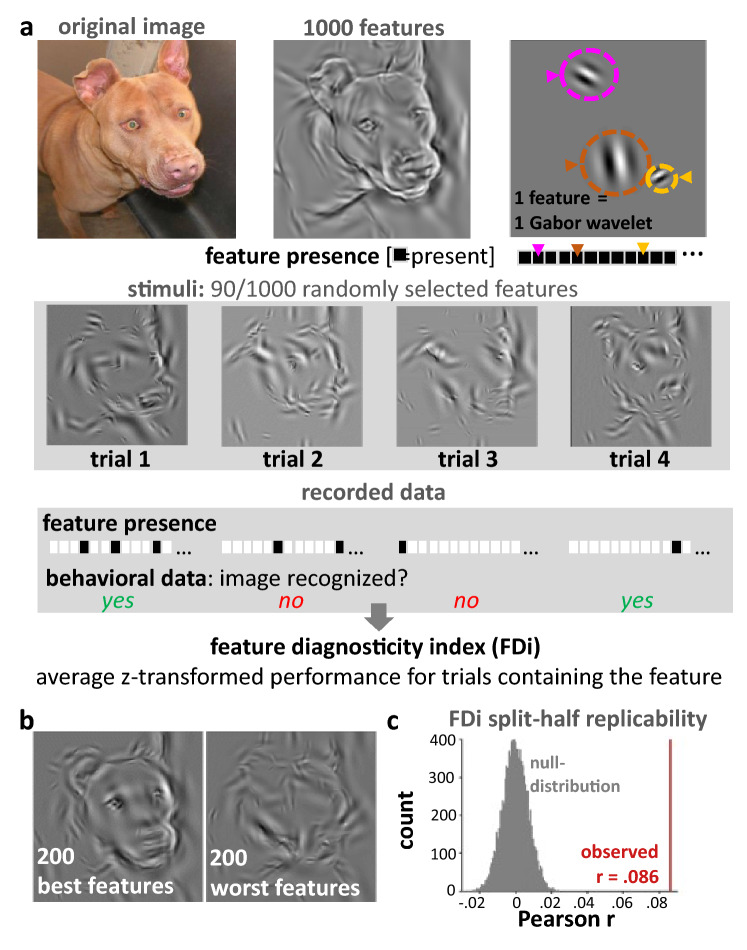
Feature diagnosticity mapping. (**a**) Stimuli were reconstructed using the 1,000 Gabor wavelets that best explained the pixel intensity in the original images. A random subset (90) of these features was presented during each trial while participants judged whether the image depicted a cat or a dog. A feature diagnosticity index (FDi) was computed based on the behavioral responses using an approach similar to reverse correlation. (**b**) Images depicting the sum of the 200 features with highest (best features) and lowest (worst features) FDi values. (**c**) graph showing that FDi value patterns replicate between participants (see “Methods” for more details).

We developed a new experimental paradigm to measure the relative contribution of low-level image features to image recognition, using a technique similar to reverse correlation^32,33^. During the experiment, 52 participants were presented with partial reconstructions of five cat and five dog images. To create these stimuli, we first selected 1,000 Gabor wavelets (with varying position, spatial frequency and orientation) which, when summed, provided a good estimate of pixel intensity values of the original cat and dog images which one can easily recognize as a dog or cat image (sums of all features are displayed in the Supplementary figure). Partial reconstructions contained a random selection of 90 of the 1,000 features (Fig. 1). Via button presses, participants indicated whether they recognized a dog, a cat or whether they were not sure.

AQ-scores of the participants were all within the neurotypical range (< 32) and ranged from 5 to 30 (M = 14.3, SD = 5.9). This range of AQ scores is somewhat lower than the typically reported AQ score range^34^ which can be explained by the fact that our participants were predominantly female (41 female, 11 male). In this context, it is worth noting the AQ test has been primarily been validated for ASD patient groups that were predominantly male^24^. Participants were assigned to the high AQ group (n = 25) if they had an autistic-spectrum quotient^24^ (AQ) higher than the median across all participants (AQ > 14) while the others were assigned to the low AQ group (n = 27). We opted for this AQ cut-off approach because it enabled us to include all participants in the analysis and because the outcome of this analysis type, in contrast to a correlational analysis, depends less on hard-to-interpret effects of small AQ-score difference. The AQ-score cut-off used during this study (AQ > 14) was comparable to the cut-offs used by previous studies using the same median-split approach^35–38^.

On average, participants successfully recognized 49.7% (SD = 15.6%) of the partial reconstructions. During these trials they either reported having recognized a cat when a cat was shown or reported having recognized a dog when a dog was shown. Note that participants, in addition to reporting that they recognized the image as a cat/dog, could also indicate that they were ‘unsure’, which they did during 29.3% of the trials (SD = 22.9%). During the remaining 21.0% of trials participants reported seeing a different animal than displayed. As a result, recognition performance reported here should not be confused with recognition performance for two-alternative forced choice paradigms. A repeated-measure ANOVA revealed that there was no difference in recognition performance between cat and dog images (50.9% and 48.4% respectively, F(1, 100) = 0.50, p = 0.48), no effect of AQ group on recognition performance (high AQ group: 47.4%; low AQ group: 51.9%; F(1, 100) = 2.49, p = 0.12), nor an interaction between these two factors (F(1, 100) = 1.23, p = 0.27).

To quantify the relative importance of visual features we computed a feature diagnosticity index (FDi) for all 10,000 features (10 images with 1,000 features each) based on the average recognition accuracy for trials containing each feature. This value, however, is affected by variance due to differences in the recognizability between images and by overall performance differences between participants (across all images and image features). This variance, however, is undesirable because we are only interested in the relative contribution of features to image recognition within each image and within each participant. Therefore, we z-scored FDi values within each participant and image (across the 1,000 features of each image). If FDi values truly reflect feature importance for image recognition, images resulting from the summation of features with the highest FDi values should be most recognizable. Visual inspection of images reconstructed from the 200 features with the highest FDi values revealed that these images were easier to recognize than images reconstructed from the 200 features with the lowest FDi values (shown for one exemplary image in Fig. 1c and for all images in the Supplementary figure). This observation, however, does not quantitatively validate the efficacy of our method. Therefore, we have, in addition, validated our method by assessing the replicability of the pattern of FDi values (across all 10,000 features) between participants. This analysis revealed significant replicability of FDi patterns across participants (Pearson r = 0.081, p < 0.0001, permutation-based test, Fig. 1c).

**Figure 2:**
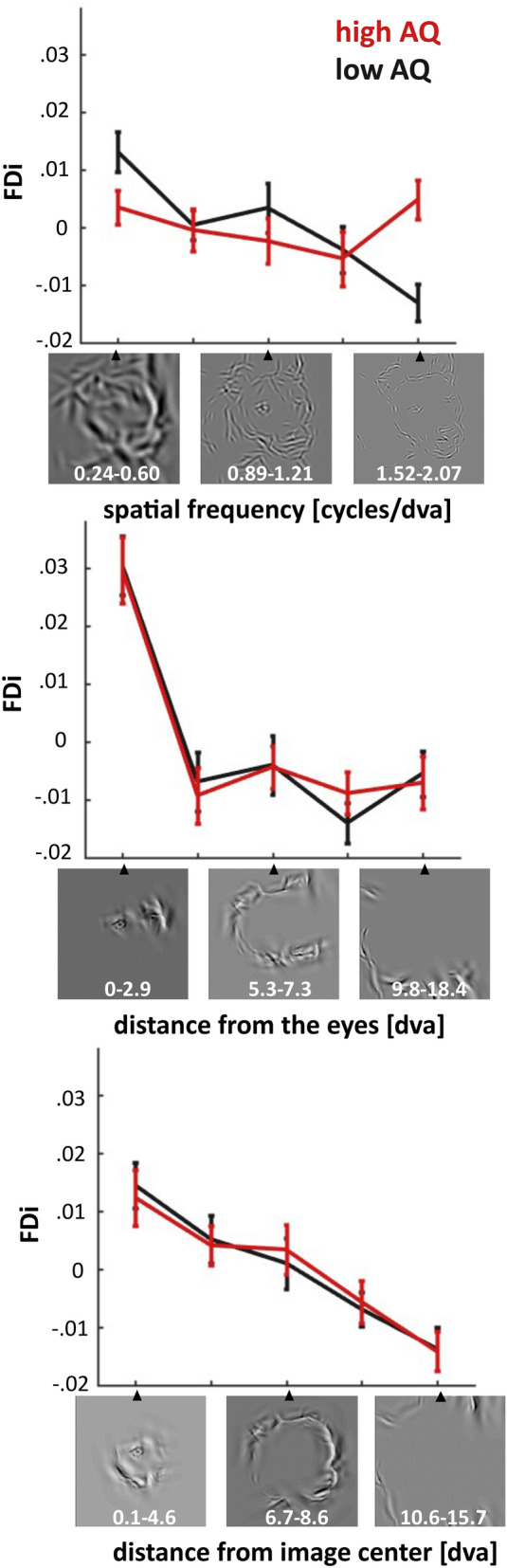
Effect of the number of autistic-like traits on the use of different types of visual features. The top panel depicts how feature diagnosticity is affected by AQ group (high AQ = AQ > 14, low AQ = AQ < 15) and feature spatial frequency. A clear interaction was observed between these two factors, which revealed that high-AQ individuals rely more on high spatial frequency information when recognizing cat and dog images. We also assessed the importance of the distance of features from the eyes (middle panel) and the image center (lower panel) but found no interaction between these factors and AQ group. Cycles/dva stands for cycles per degree visual angle.

After having established that FDi values measure the importance of features for image recognition, we tested if FDi values are elevated for high-spatial frequency features in high-AQ individuals. To this end, we grouped features into five equally-sized bins (containing 2000 features each) with ascending feature spatial frequencies. Spatial frequency ranges for bin 1–5 in cycles per degree visual angle were resp.: 0.24–0.60, 0.60–0.89, 0.89–1.21, 1.21–1.52 and 1.52–2.07. We tested for an interaction between spatial frequency and AQ group using a repeated-measure ANOVA with average bin FDi values as the dependent variable. This analysis revealed a main effect of spatial frequency (F (4, 250) = 3.97, p < 0.005, Fig. 2), and an interaction between spatial frequency and AQ group (F (4, 250) = 4.12, p < 0.005, Fig. 2). The interaction was mostly driven by the high-AQ group having elevated FDi values for the highest spatial frequency bin (0.005 and − 0.013 resp.; t(50) = 3.80, p < 0.001). Therefore, our results are consistent with high-AQ individuals relying more on local details for image recognition.

Previous behavioural studies have linked ASD to reduced gaze durations for the eye-region in human faces^28–30^, and increased gaze durations for the central area of images^31^. To assess this, we repeated the analysis while grouping features into five ascending bins (containing 2,000 features each) according to feature distance from the nearest eye (bin 1–5 resp. ranged from 0° to 2.9°, 2.9°–5.3°, 5.3°–7.3°, 7.3°–9.8° and 9.8°–18.4° visual angle) or distance from the image centre (bin 1–5 resp. ranged from 0.1° to 4.6°, 4.6°–6.7°, 6.7°–8.6°, 8.6°–10.6° and 10.6°–15.7° visual angle). This analysis revealed two main effects which indicate that FDi decreases as a function of the distance from the nearest eye (F (4, 250) = 27.4, p < 0.00001, Fig. 2) and distance from the image centre (F (4, 250) = 14.3, p < 0.00001, Fig. 2). Importantly, neither of these effects was modulated by AQ group. Therefore, our data does not replicate previously reported ASD effects on image centre and eye-region processing. It is important to note that in contrast to these previous studies, our results are not based on eye movements nor on data from clinically diagnosed ASD patients.

**Figure 3:**
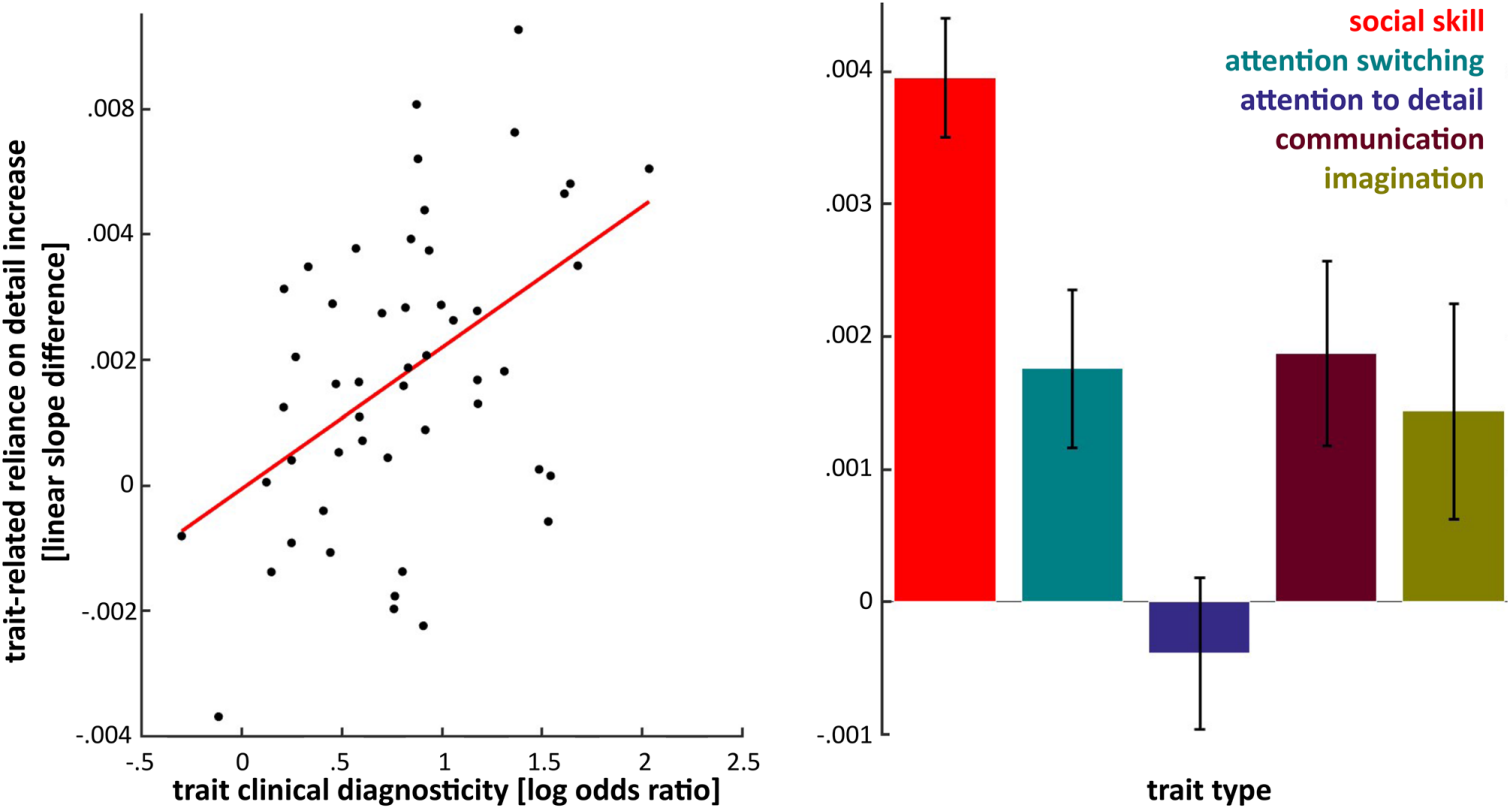
Reliance-on-detail effects scale with the clinical relevance of autistic-like traits. We assessed how a positive score on each of the 50 AQ items (referred to as ‘traits’) increased our participants preference for high spatial frequency features. Reliance-on-detail was quantified for each participant as the linear regression coefficient between their average FDi values for each spatial frequency bin and the ascending bin numbers (1–5, see Fig. 2). Clinical diagnosticity of traits were quantified as the (natural) log of the trait prevalence ratio between clinically diagnosed ASD individuals and neurotypical students^24^. The scatter plot shows that the clinical diagnosticity of traits scales positively with how much the presence of traits increases reliance-on-detail (left). Furthermore, reliance-on-detail increases were found to depend on the trait type (right), with the presence of ‘social skill’ traits leading to above-average increases and ‘attention to details’ traits to below-average increases. Note here that the trait types labeled as ‘social skill’, ‘attention switching’, ‘communication’ and ‘imagination’ all refer to difficulties in the respective domains while ‘attention to detail’ refers to its domain positively.

Does our finding of an increased reliance-on detail for visual recognition in high AQ individuals generalize to individuals with an ASD diagnosis? Our study does not provide direct evidence for this as we measured a neurotypical student population. However, we are able to provide indirect evidence by testing if visual-detail-reliance depends most on the presence of clinically diagnostic AQ traits (with ‘trait’ we refer to a positive score on one of the 50 items of the AQ questionnaire). To this end, we quantified the clinical diagnosticity the 50 autism traits as the (natural) log of the trait prevalence ratio between clinically diagnosed ASD individuals and neurotypical students, making use of previously published prevalence data^24^. For example, the highest log odds ratio (2.04) was assigned to the trait measured with the item “I enjoy social occasions”, which ASD individuals disagree with 7.7 times more often than neurotypical students. In addition, we quantified the reliance-on-detail for each participant as the linear regression coefficient between their average FDi values for each spatial frequency bin and the ascending bin numbers (1–5, see Fig. 2). Subsequently, we performed a robust regression analysis that confirmed our hypothesis by revealing that our reliance-on-detail measure was best predicted by the presence of the most clinical diagnostic autistic traits (slope = 0.0023, t(48) = 3.40, p < 0.005, Fig. 3).

In addition, we assessed how trait-related increases of reliance-on-detail depended on the five different trait types (social skill, attention switching, attention to detail, communication and imagination) that the AQ questionnaire was designed to measure (with ten items for each type of ASD trait)^24^. Note here that the trait types labelled as ‘social skill’, ‘attention switching’, ‘communication’ and ‘imagination’ all refer to difficulties in the respective domains while ‘attention to detail’ refers to its domain positively. A one-way ANOVA revealed an effect of trait type (F(4, 45) = 5.89, p < 0.001). Five post-hoc t-tests (Bonferroni corrected) testing for a difference between each trait type versus all others revealed that the presence of ‘social skill’ traits increase reliance-on-detail more than all other trait types (t(48) = 3.72, p < 0.005) while ‘attention to detail’ traits give rise to a below-average effect (t(48) = − 3.48, p < 0.01). Therefore, our findings suggest that enhanced reliance on detail for image recognition is most predictive for autistic traits related to self-reported social difficulties.

Finally, we analysed reaction times (M = 895 ms, SD = 313 ms). We found that high-AQ participants took longer to respond than low-AQ participants (993 ms vs 804 ms, t(50) = 2.25, p < 0.05). Unfortunately, reaction times were found to carry no information about the relative importance of visual features for image recognition as reaction time based FDi values did not replicate across participants (Pearson r = 0.0077, p = 0.14, permutation-based test).

## Discussion

In summary, we developed a method that measures the relative contribution of low-level visual features to image recognition. With this method, we obtained evidence for natural image recognition depending more on high spatial frequency features in individuals with an above-median number of autistic traits. Therefore, the presence of a greater number of autistic traits appears to predict enhanced reliance on fine details for natural image recognition. This effect was found to be driven most by the presence of autistic traits with the highest clinical relevance, which increases the likelihood that our main finding generalizes to clinically diagnosed ASD individuals.

Our study sheds new light on the ongoing debate regarding whether a bias towards processing details underlies ASD-related social deficits^4,9,10,13–16,39^ as we find that reliance on details is best predicted by positive scores for AQ items from the AQ subscale measuring self-reported social difficulties. Therefore, in contrast to findings from a fairly large body of research^10,13–16,39^, our results suggest that social deficits central to ASD might be related to having an eye for detail. Our findings, however do converge with a recent study demonstrating that performance for the embedded figure task is positively related to scores for items of the same social skill AQ-subscale^4^. In this context, it is important to note that previous research indicates that all AQ-subscales, except for the ‘attention to detail’ subscale, reflect a single general ASD attribute, thought to be related to difficulties in social interactions^40^, rather than specific ASD symptoms^41^. Consistent with this, we found that scores for all subscales, except for ‘attention to detail’, were positively associated with our measure of detail-focused visual information processing. Furthermore, we would like to point out that the ‘attention to detail’ AQ subscale contains items measuring attention towards both perceptual and more abstractly defined details (e.g. item 32: “I notice patterns in things all the time.”), which could explain why we observed no relationship between scores for this subscale and detail-focused visual information processing.

In sum, our findings suggest that original proposal of detail-focused processing underlying the social deficits associated with ASD^8^ appears to be worth revisiting. An important limitation of our study, however, is that our study is restricted to comparing groups of neurotypical participants with above- and a below median AQ scores. Despite the fact that this comparison has proven to be extremely useful for modelling ASD^42^, an ultimate verification of a relationship between detail-focused visual information processing during natural image recognition and ASD-related social difficulties would require replication of our findings for a clinically diagnosed ASD population.

If ASD is associated with image recognition being driven more by details, what could be the underlying neural mechanisms? One possibility is that individuals with ASD process visual information differently already at an early stage of cortical visual information processing, e.g. already within the primary visual cortex^19^. This would be compatible with the finding that ASD individuals appear to have enhanced sensitivity for high-spatial frequency gratings^20^ and with the finding of early (125 ms post stimulus) enhanced EEG responses to high spatial frequency information in 4-year old children with ASD^43^. A second possibility—that is compatible with reports suggesting that ASD does not affect elementary vision^22,44^—would be that the reliance-on-detail effect reported here results from higher level brain areas responsible for object recognition, e.g. the inferior temporal cortex, being more reliant on lower-level representations of high-spatial frequency features. Differentiating between these two possible underlying neural mechanisms will require neuroimaging studies aimed at revealing whether ASD enhances the extent to which high-spatial frequency information is encoded in the primary visual cortex and/or if ASD is associated with enhanced transmission of high-spatial frequency visual information to higher level brain areas.

In contrast to previous eye-tracking studies^29–31^, we found no evidence for ASD-related reduced processing of the eye-region, nor an increased processing of the central area of images. One possible explanation for this discrepancy might be the fact that we investigated ASD by comparing individuals with above- and below-median AQ scores instead of comparing clinically diagnosed ASD individuals with healthy controls. Another intriguing possible explanation could be that ASD leads to elevated gaze durations to the image centre and eye-region, but that this does not translate into object recognition relying more on the features inside these areas. To test the latter, future studies will need to combine our novel psychophysical paradigm with eye-tracking.

In conclusion, our results show that natural image recognition is driven more by visual details in neurotypical individuals with an elevated number of autistic traits. Given that the most clinically relevant autistic traits best predict this effect, this finding is suggestive for a detail-focused information processing bias being a ubiquitous property of vision in ASD individuals. In addition, this detail-focused processing bias was found to predict increased self-reported social difficulties. Therefore, we propose that the original proposal of WCC^8^ that enhanced local information processing underlies the social deficits associated with ASD might be worth revisiting.

## Methods

### Participants

52 healthy student volunteers (11 male, 41 female: average age = 19.4, SD = 1.05) with normal or corrected-to-normal vision took part in this experiment. All participants gave their informed consent after being introduced to the experimental procedure in accordance with the Declaration of Helsinki. The experimental procedure was approved by the ethics committee of the University of Birmingham (ethics reference ERN_15-1374P). Participants all filled out the 50-item Autism Spectrum Quotient (AQ) questionnaire for adults^24^ and were assigned to the high- and low AQ groups depending on whether their AQ scores exceeded the median AQ score for all participants (AQ > 14).

### Stimuli

Portrait images of five cats and five dogs were converted to 250 × 250 grey-scale images. We then used a custom-made algorithm, implemented in Matlab 2016a, to reduce images to 1,000 Gabor wavelets. The aim of this algorithm was to find a set of wavelets that is able to describe most of the coarse and fine image details (see Fig. 1a for a visualization of the features selected for one of the images). Wavelets considered had 29 (*n* = [1, 2, …, 29]) exponentially increasing spatial frequencies (*sf*) between 0.24 and 2.07 cycles/visual degree angle:

$$sf=\frac{10}{45}\times {1.08}^{n}$$

Wavelets were considered with 18 equidistant orientations between 0° and 180° (0°–170° in steps of 10°). Features were selected iteratively from the lowest to the highest spatial frequency. During the first iteration, wavelets were selected based on the original grey-scale image. For consecutive iterations, the input image was a residual image resulting from least-square regression of the input to the previous iteration while using previously selected wavelets as regressors. During each iteration, eighteen Gabor wavelet filters (one per orientation) were applied to the input image. The output of this analysis enabled us to find the optimally-fitting orientation and phase for Gabor wavelets cantered on each pixel of the input image for the current iterations’ spatial frequency. From these 250^2^ wavelets, we considered only 25% with the highest amplitude. From these wavelets, we then randomly selected a number of wavelets (*nw*) increasing as a function of spatial frequency:

$$nw={272 \times sf}^{1.8}$$

The exact parameters of this formula were determined exploratively based on how well the sum of all selected wavelets captured all coarse and fine visual details of the original images (based on visual inspection). This resulted in 21 features being selected for 0.24 cycles/visual degree angle wavelets and 1,008 features for 2.07 cycles/visual degree angle wavelets. During each iteration, we computed the covariance of each feature with the input image and discarded wavelets with covariances smaller than a fifth of the maximum observed covariance value. Finally, we selected 1,000 wavelets from all spatial frequencies (from all iterations) having the highest covariance with the original grey-scale image. Amplitudes where set to an equal value for all wavelets. Partial reconstructions of the images were created by randomly selecting 90 wavelets from the set of 1,000 and summating them (see Fig. 1a). The pixel intensity range of the resulting images was kept constant, covering the full 0–255 range.

### Experimental procedure

Participants viewed partial reconstructions on an LCD from 70 cm distance [visual degree angle (°), horizontal and vertical dimensions of the screen: 51.6° × 30.4°]. Each trial started with a grey screen and a central fixation cross which participants were instructed to fixate, which we presented for a duration between 250 and 400 ms. Afterwards, a partial reconstruction was presented on a central area of the screen (covering 22.5° × 22.5°) and remained on the screen until a button press was made. Participants used their right hand to press one of the three available buttons with which they indicated having recognized a cat, a dog or that they weren’t sure about the type of animal they were shown. The next trial started as soon as a button was pressed. Due to the self-paced nature of the paradigm, participants completed a variable number of sessions (6, 5, 4, 3 and 2 sessions were completed by 2, 14, 25, 10 and 1 participants resp.), which each consisted of 50 trials per image. Stimuli were presented and behavioural data was recorded using Matlab 2016a and the PsychToolbox^45^ (version 3).

### Data analysis

First, we assessed for each trial whether the image depicted was recognized. Then, we computed the average recognition performance for each participant and image feature by computing the average performance for trials containing the respective feature. This provided us with a three-dimensional matrix of recognition performances with the dimension number of participants (52), number of images (10) and number of features (1,000). Next, we obtained our feature diagnosticity index (FDi) values by z-transforming the performance values within each participant and image. This final step is important because it precludes FDi values being higher for features of images that are easier to recognize. Furthermore, it ensures that participants’ relative contributions to the following analyses do not depend on their average recognition performance nor the variability of their responses.

To evaluate the replicability of the observed FDi values, we randomly split the data into two halves (two times 26 participants) 100 times and computed the Pearson correlation between the average FDi values across splits. Replicability was then measured as the average Pearson correlation value across these 100 splits. The probability of observing this value by chance was determined by computing a null-distribution by re-computing this value 10,000 times while permuting the relative feature labels across splits.

To assess effects of spatial frequency, distance from the nearest eye and distance from image centre on FDi values, we created five equally sized ascending bins based on each of these parameters. Thereafter, we computed the average FDi value within each of these bins separately for each participant. In addition, we assigned each participant to the high AQ group and low AQ group depending on whether their AQ was or was not higher than the median AQ across all participants. This enabled us to perform three 5 × 2 repeated measure ANOVAs with average within-bin FDi values as the dependent variable. Each ANOVAs second factor was AQ group while the first factor was the binning feature: spatial frequency, distance from the nearest eye or distance from image centre.

## Supplementary information

Supplementary figure.

## Data Availability

Behavioural data and Matlab code for our data analyses can be downloaded from the online public GitHub repository: https://github.com/arjenalink/AQ_EyeForDetail_NSR.
