# Supplementary material for: Clinically relevant autistic traits predict greater reliance on detail for image recognition
Source: Sci Rep. 2020 Aug 28;10:14239. doi: 10.1038/s41598-020-70953-8 (PMC7455566; doi:10.1038/s41598-020-70953-8)
Supplement: Supplementary file 1 — Supplementary figure. [file 41598_2020_70953_MOESM1_ESM.pdf]

Clinically relevant autistic traits predict greater reliance on detail for image recognition

Arjen Alink[1] *& Ian Charest*[2]

Supplementary Information

[1]*University Medical Centre Hamburg-Eppendorf, Germany*

[2]*University of Birmingham, United Kingdom*

Corresponding author

Arjen Alink

a.alink@uke.de

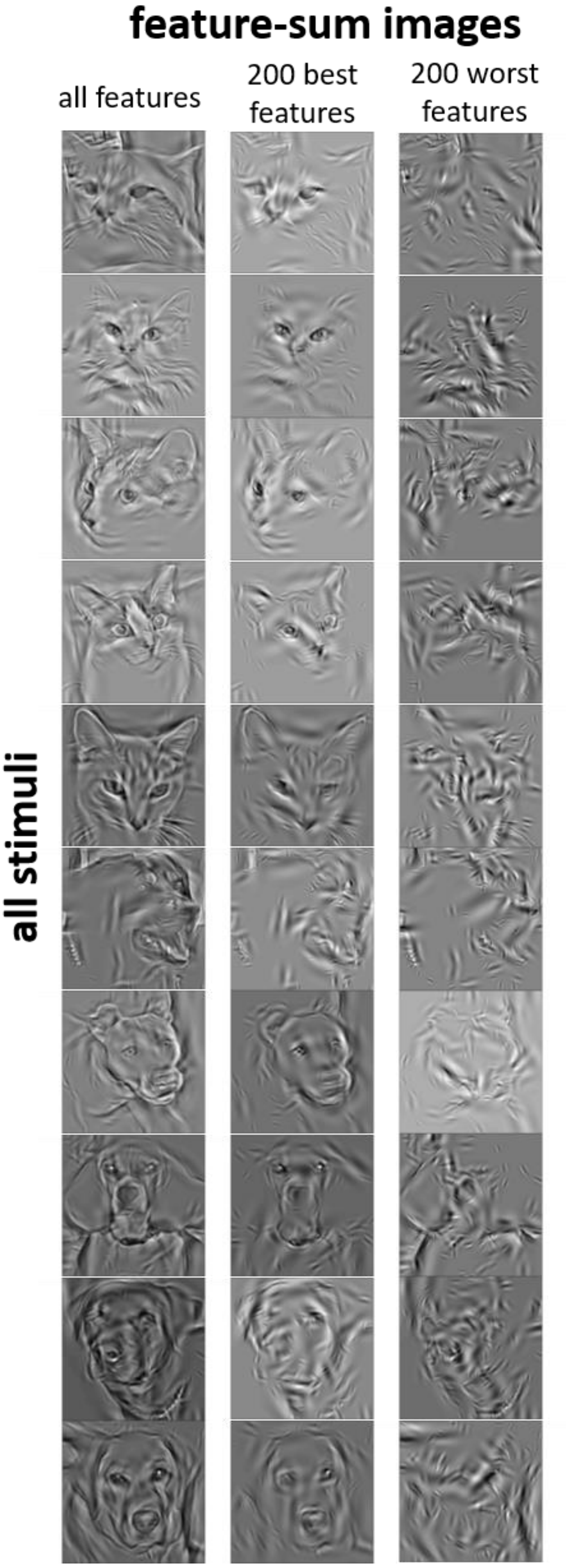

Supplementary figure showing the sum of all 1000 Gabor wavelet features for all 10 images used during the experiment and the 200 best and worst features based on their associated FDi values (across all participants)
